# Real-time in vivo tracking of nanoparticulate vaccines in inguinal lymph nodes

**DOI:** 10.1186/s41120-026-00163-5

**Published:** 2026-04-27

**Authors:** Bishal Misra, Kaitlyn M. Landreth, William H. Pentz, Tracy W. Liu, Sharan Bobbala

**Affiliations:** 1 Department of Pharmaceutical Sciences, West Virginia University, Morgantown, WV 26506 USA; 2 Department of Microbiology, Immunology and Cell Biology, West Virginia University, Morgantown, WV 26506 USA; 3 West Virginia University Cancer Institute, Morgantown, WV 26506 USA

**Keywords:** Lipid nanoparticles, Lymph nodes, Ionizable lipids, mRNA vaccines, Intravital imaging, Window chamber

## Abstract

Understanding the kinetics of nanoparticle vaccine drainage to the lymph nodes and their residence time in these tissues is critical in vaccine development. Evaluating these parameters using conventional techniques is expensive, time-consuming, and often leads to variable data, as several animals at different time points need to be sacrificed, and lymph nodes need to be harvested for characterization. Here, we adapted an abdominal window chamber for intravital imaging of the lymph nodes, enabling real-time tracking of ionizable lipid nanoparticles (LNPs) and polymeric nanoparticles in live mice following intramuscular administration. To mimic delivery of vaccine payloads, these nanoparticles were encapsulated with luciferase mRNA or a small molecule fluorophore, indocyanine green, and tracked for bioluminescence and fluorescence, respectively, in the lymph nodes. The results demonstrated differential lymph node drainage kinetics and retention for LNPs and polymeric nanoparticles. Notably, variations in the ionizable lipid components of LNPs resulted in changes in the mRNA translation ability within the lymph nodes. Taken together, this study introduces a valuable tool to optimize nanoparticle vaccine design in preclinical studies.

**Graphical Abstract:**

**Figure:**
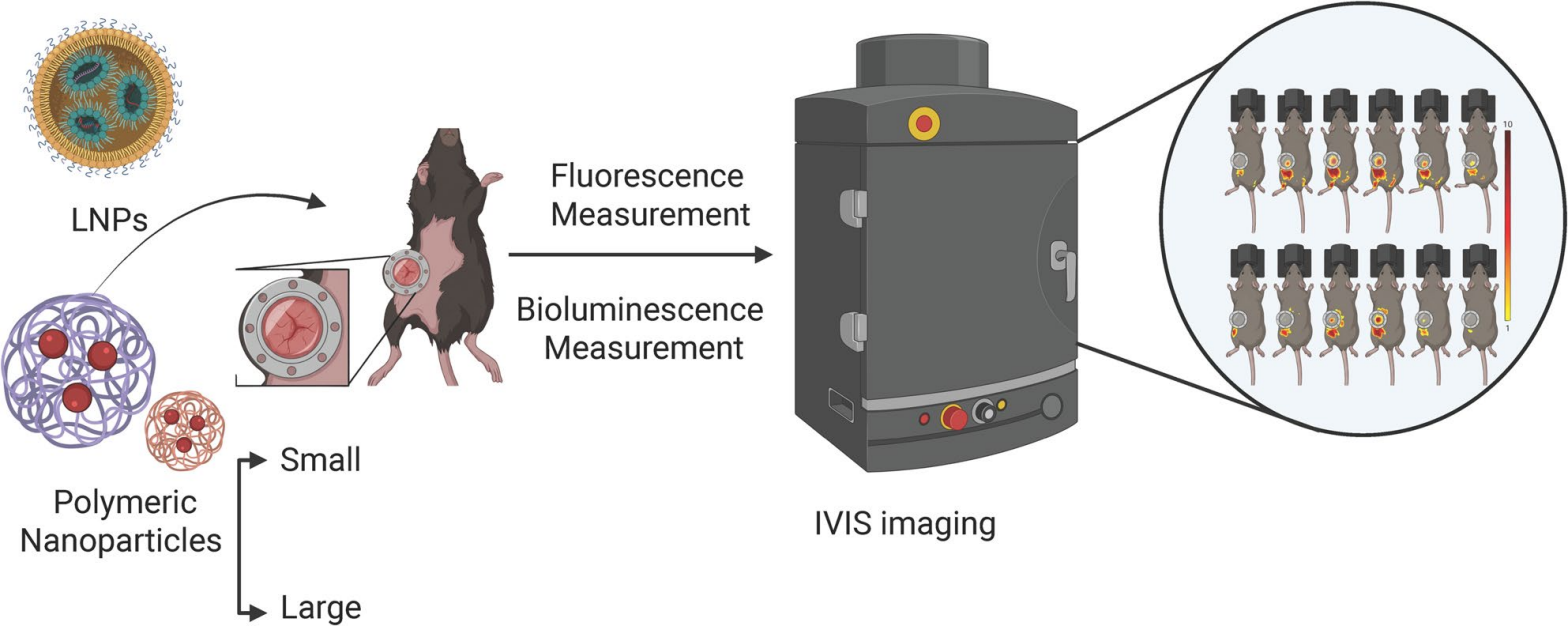
Real-time in vivo tracking of the lymph node drainage kinetics of nanoparticles encapsulating mRNA or small molecule fluorescent dye, facilitated by the use of a lymph node window chamber. Created in BioRender. https://BioRender.com/786dnai.

**Supplementary Information:**

The online version contains supplementary material available at 10.1186/s41120-026-00163-5.

## Introduction

Nanoparticles are indispensable for encapsulating diverse antigens or adjuvants and efficiently delivering them to antigen-presenting cells to induce strong immune responses. Nanoparticle vaccines are either encapsulated with antigenic components such as mRNA, subunit peptides, and recombinant proteins or small-molecule adjuvants. Of note, nanoparticles alone demonstrate adjuvant activity due to their particulate nature (Jiang et al. 2025). Based on physicochemical characteristics such as shape, size, and nature of the material, nanoparticle drainage to the lymph nodes is mediated either directly through lymphatic vessels or indirectly through immune cells at the administration site. For instance, nanoparticles smaller than 30 nm can drain into lymph nodes through lymphatic transport, whereas those larger than 100 nm are drained to lymph nodes through immune cells (Hassett et al. 2021). Due to their pathogen-mimicking nature and enhanced interaction with antigen-presenting cells, lipid-based and polymeric nanoparticles have been widely studied for prophylactic and therapeutic vaccine applications (Chattopadhyay et al. 2017; Grego et al. 2021). Of note, the recent clinical success of COVID-19 mRNA vaccines was also possible using lipid nanoparticles (LNPs), which enhanced the stability and allowed precise delivery of mRNA to immune cells.

COVID-19 vaccines, mRNA-1273/SPIKEVAX (Moderna) and BNT162b2/COMIRNATY (BioNTech/Pfizer) utilize the most advanced ionizable LNP strategy specifically designed for the delivery of nucleic acids such as mRNA and siRNA. Most recently, Moderna’s respiratory syncytial virus vaccine, mRESIVIA, was approved by the FDA, which also employs the LNP technology. Ionizable LNPs typically contain four components, including ionizable lipid, phospholipid, PEGylated lipid, and cholesterol. Specifically, the ionizable lipid component allows LNPs to remain neutral at physiological pH and protonated at endo-lysosomal environment (pH 4–5), facilitating endosomal disruption and nucleic acid release inside immune cells (Han et al. 2021; Swetha et al. 2023). Currently, several different types of ionizable LNPs are under preclinical development for the effective delivery of mRNA vaccines to combat infectious diseases and cancers. In contrast, polymeric nanoparticles are valued for their customizability, enabling them to mimic various pathogens, microbes, and viruses (Wibowo et al. 2021). Common polymeric nanoparticles include solid particles synthesized from natural polymers such as chitosan and alginate, or synthetic polymers including polylactic acid (PLA), Poly(lactic-co-glycolic acid) (PLGA), and poly(ε-caprolactone) (PCL) (Wibowo et al. 2021). Furthermore, micellar polymeric nanoparticles, polymersomes, and polymeric nanogels have also been investigated for vaccine applications requiring controlled release of antigen or adjuvant, targeted delivery to immune cells, and enhanced immune responses (Grego et al. 2021; Ho et al 2021; Wibowo et al 2021).

Since adaptive immune responses are primarily initiated in lymphoid organs, the transport of vaccine components from the injection site to lymph nodes is critical for effective vaccination (Bachmann et al. 2010). Consequently, monitoring the drainage kinetics and residence of vaccine nanoparticles in lymph nodes is essential in the development and optimization of efficacious vaccine formulations. For example, the success of an mRNA vaccine can be gauged through efficient drainage of LNPs to lymphoid organs and translation of mRNA into an antigenic protein inside immune cells. It is important to note that different ionizable LNPs may demonstrate variable drainage kinetics to lymph nodes and mRNA translation. The current standard for evaluating nanoparticle vaccine drainage to the lymph node is heavily reliant on extensive in vivo studies, which include incorporating multiple animals for each time point and the sacrifice of a large group of animals, followed by the harvesting of organs for characterization. Of note, ex vivo IVIS imaging of harvested lymph nodes or flow cytometry analysis of single-cell suspensions of lymph nodes are often employed in characterizing the efficiency of nanoparticle vaccine delivery. These practices can delay the optimization timelines of formulations in the discovery phase and may also lead to variable data. There is an urgent requirement for the development of characterization tools that can track nanoparticle vaccine drainage to lymph nodes in real-time in vivo with high precision.

Here, we employed a lymph node window chamber integrated with IVIS imaging to track the lymph node drainage and retention kinetics of nanoparticles encapsulating mRNA or a small molecule payload in a live animal following their administration. We validated this model using clinically relevant ionizable lipid nanoparticles that are employed in COVID-19 mRNA vaccines and polymeric nanoparticles with variable sizes. These nanoparticles were either encapsulated with luciferase mRNA or small molecule fluorophore payload (indocyanine green) and tracked for their bioluminescence and fluorescence, respectively, using IVIS imaging. Taken together, this study introduces an exciting methodology to optimize nanoparticle vaccine formulations to achieve the desired immune responses.

## Materials and methods

### Materials

PLGA10K-PEG2k-Mal was procured from Nanosoft Polymers (Winston-Salem, NC). D-α-Tocopherol polyethylene glycol 1000 succinate (TPGS) was sourced from Millipore Sigma (Burlington, MA). All the lipids and Indocyanine Green (ICG) was acquired from Cayman Chemical (Ann Arbor, MI). Tetrahydrofuran Anhydrous (THF) was obtained from Tokyo Chemical Industry Co.(Portland, OR). Phosphate Buffered Saline was obtained from Gibco (Waltham, MA). SnakeSkin™ Dialysis Tubing was procured from Thermo Fisher Scientific (Waltham, MA). Sephadex^®^ LH-20 Size Exclusion Resin was purchased from Cytiva (Marlborough, MA). Firefly luciferase mRNA was obtained from APExBIO Technology LLC (Houston, TX). TNS reagent (6-p-toludino-2-napthalene-sulfonic acid) was procured from Abcam (Cambridge, United Kingdom).

### Formulation of nanoparticles

SM102 and ALC0315 ionizable lipid nanoparticles were fabricated using the Flash nanoprecipitation (FNP) technique as previously described (Misra et al. 2024, 2025). Briefly, lipids were dissolved in ethanol with varying molar ratios. For SM102 LNPs, a 50:10:38.5:1.5 molar ratio of ionizable cationic lipid (SM102), 1,2-Distearoyl-sn-glycero-3-PC (DSPC), cholesterol, and PEGylated-lipid (DMG-PEG2000), respectively was used. For ALC0315 LNPs, a ratio of 46.3:9.4:42.7:1.6 of ionizable cationic lipid (ALC0315), DSPC, cholesterol, and PEG lipid (ALC0159), respectively, was employed. A solution of 0.5 mg ICG in methanol was added to the organic phase containing the lipids. Firefly luciferase (FFLuc) mRNA was dissolved in a 50 mM sodium citrate buffer at pH 4. The aqueous and organic phases were then impinged in the confined impingement jet (CIJ) mixer, and the LNPs were collected in a 2 mL reservoir with sodium citrate buffer (pH 4). For polymeric nanoparticles, the organic phase containing either a mixture of 20 mg TPGS and 0.3 mg ICG in 500 µL of methanol or 0.3 mg of ICG and 20 mg of PEG-PLGA in 500 µL 5:8 methanol: THF solution was prepared. These organic phases were impinged against aqueous phase water in a CIJ mixer and collected into a 2 mL water reservoir. All LNP and polymeric nanoparticle formulations were then dialyzed against sterile PBS overnight. Subsequently, the formulations were filtered using an LH20 column to remove free ICG dye.

### Characterization of the nanoparticles

The nanoparticles’ hydrodynamic size and surface charge were assessed using dynamic light scattering (DLS) and electrophoretic light scattering (ELS) with the Zetasizer Ultra from Malvern Instruments, UK. Phosphate-buffered saline (PBS) served as the dispersant. The apparent pKa of the LNPs was measured using the TNS assay (Misra et al. 2024). The encapsulation efficiency of FFLuc mRNA in LNPs was measured using the Promega QuantiFluor RNA system. ICG was quantified by measuring the fluorescence (λEx = 790 nm; λEm = 830 nm) using a SpectraMax microplate reader.

Negative stained Transmission electron microscopy (TEM) imaging was performed using JEOL 1400 with AMT-Nanosprint15L-MkII camera. LNPs were applied to UV-treated, carbon-coated grids (Ted Pella, 01840-F) and stained immediately with 1% aqueous uranyl acetate before taking the images. The samples were wicked dried with filter paper before imaging.

### Lymph node window chamber implantation surgery

All the animal studies were performed after approval from the Institutional Animal Care and Use Committee (IACUC) at West Virginia University (protocol #: 2109047227). All methods performed in this study were in accordance with the IACUC guidelines and regulations. C57BL/6J mice (The Jackson Laboratory, ME, USA) between 8 and 14 weeks old were used for window chamber implantation. Mice were anesthetized using 2% isoflurane in oxygen (2 mL/min flow rate) and the hair was removed from the ventral side of the mouse, around the right hind limb. Mice received a single injection of 1 mg/kg buprenorphine XR (MWI Animal Health, Atlanta, GA, USA) prior to surgery. All surgical procedures were performed using aseptic conditions, while maintaining body temperature at physiological levels using a heating pad. The skin was prepared by three alternating washes of betadine and 70% ethanol, and an ophthalmic ointment was applied to the animal’s eyes. The toes pinch reflex test was utilized prior to incision to confirm the animals had attained surgical anesthesia. A 1 cm incision was made in the skin to the right of the midline on the ventral side of the animal. The skin was gently separated from the abdomen and the fascia, the right inguinal lymph node located, and our previously reported abdominal window chamber (Liu et al. 2021a, 2021b) was placed in the opening. The abdominal window chamber was held in place using a purse string non-resorbable suture. The lymph node was held within the window chamber using a lymph node backing (Fig. 2A) produced by the Animal Models and Imaging Core facility at West Virginia University using a 3D printer. Chamber backing design was created using Autodesk Tinkercad software (tinkercad.com). The resulting STL file was sliced using Bambu Studio version 1.10.1.50 and printed using a Bambu Lab X1 Carbon 3D printer on a 55 °C heated cool build plate. Backing was made using 0.58 g (0.19 m) matte black Bambu PLA Basic filament at 220 °C with no supports. Default printer settings included a 0.2 mm layer height, aligned seam position, and a maximum print speed of 300 mm/s. The backing was secured to the skin and the back of the abdominal window chamber at four locations using non-resorbable sutures, aligning with the pre-existing holes in both the backing and the chamber, thereby ensuring the lymph node remained positioned within the imaging window. An optically translucent silicone seal (Kwik-Sil™, World Precision Instruments, Sarasota, FL, USA) was used to fill in the window chamber and hold a 1.2 cm glass coverslip in place. Mice were subcutaneously injected with 1 mL saline to maintain hydration, were monitored until fully recovered from surgery and recovered in single-housing cages on heating pads. Once mice were fully recovered and alert post lymph node window chamber implantation, mice were injected intramuscularly (IM) with the nanoparticle formulations. Mice may be monitored for up to 14 days following lymph node window chamber placement; however, the sutures used to secure the backing may require replacement during this monitoring period.

### In vivo imaging

Macroscopic imaging occurred using the IVIS Spectrum CT (Perkin Elmer, Waltham, MA). Mice were anesthetized using 2% isoflurane in oxygen. Fluorescence imaging of the ICG-loaded nanoparticles and bioluminescence imaging of the FFLuc-loaded nanoparticles occurred at 0.5, 2, 4, 6, 24, and 48 h post IM injection of nanoparticle formulations to the quadricep muscle. The volume of each injection was 50 µL with an mRNA dose of 2.5 µg/mouse and an ICG dose of 10 µg/mouse. The total lipid and polymeric concentration in the injected formulations was 10 mg/mL. Bioluminescence imaging occurred 9 min post D-luciferin injection (150 mg/kg of body weight, i.p) using the following acquisition parameters: time, auto; binning, 8; FOV, 6.6; f/stop, 1; filter, open. Quantification of fluorescence and bioluminescence images used the Living Image Software version 4.5.2 (Perkin Elmer, Waltham, MA, USA). For each image and time point, an average background region of interest (ROI) was defined to estimate background signal. A consistent ROI encompassing the 1.2-cm coverslip area of the lymph node window chamber was then applied to quantify signal intensity. Fluorescence and bioluminescence measurements were background-subtracted using the corresponding background ROI for each time point to account for temporal fluctuations in imaging noise. Quantified signal values were log₂-transformed for graphical representation.

## Results and discussion

**Fig. 1:**
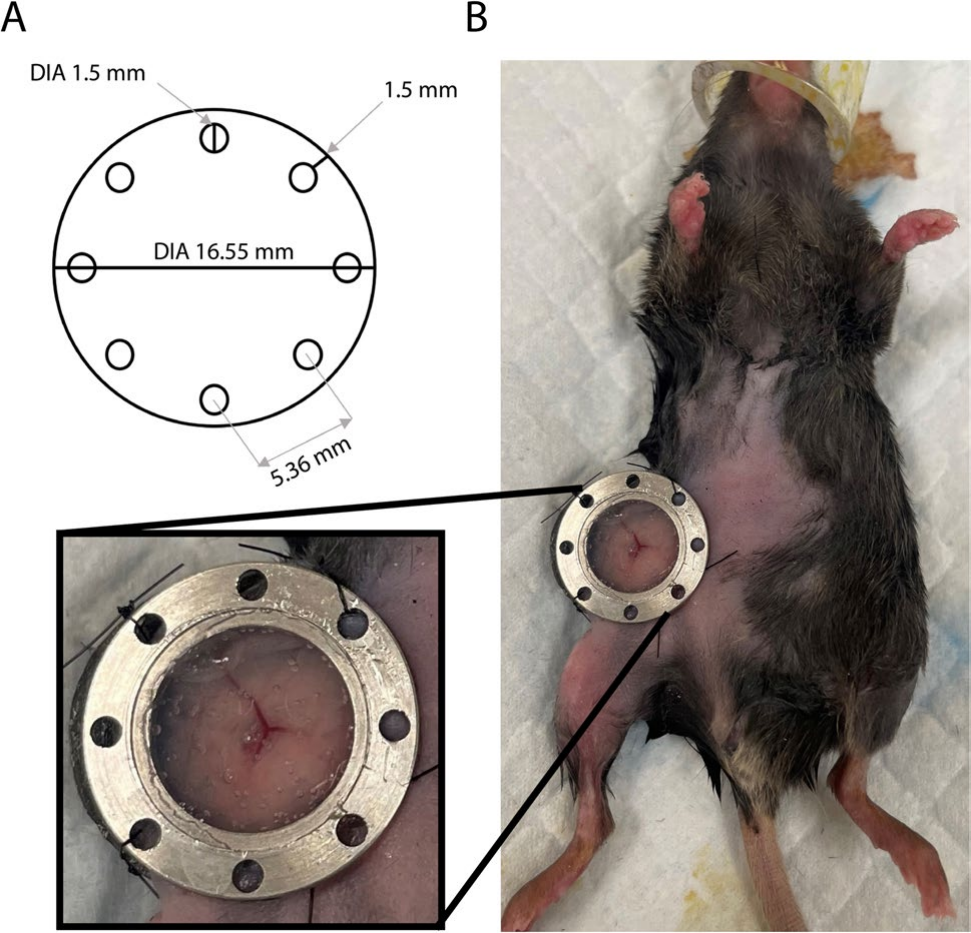
Lymph node window chamber. **A** Diagram with measurements of the lymph node backing. **B** Representative photograph of an implanted lymph node window chamber with a zoomed-in view

**Fig. 2:**
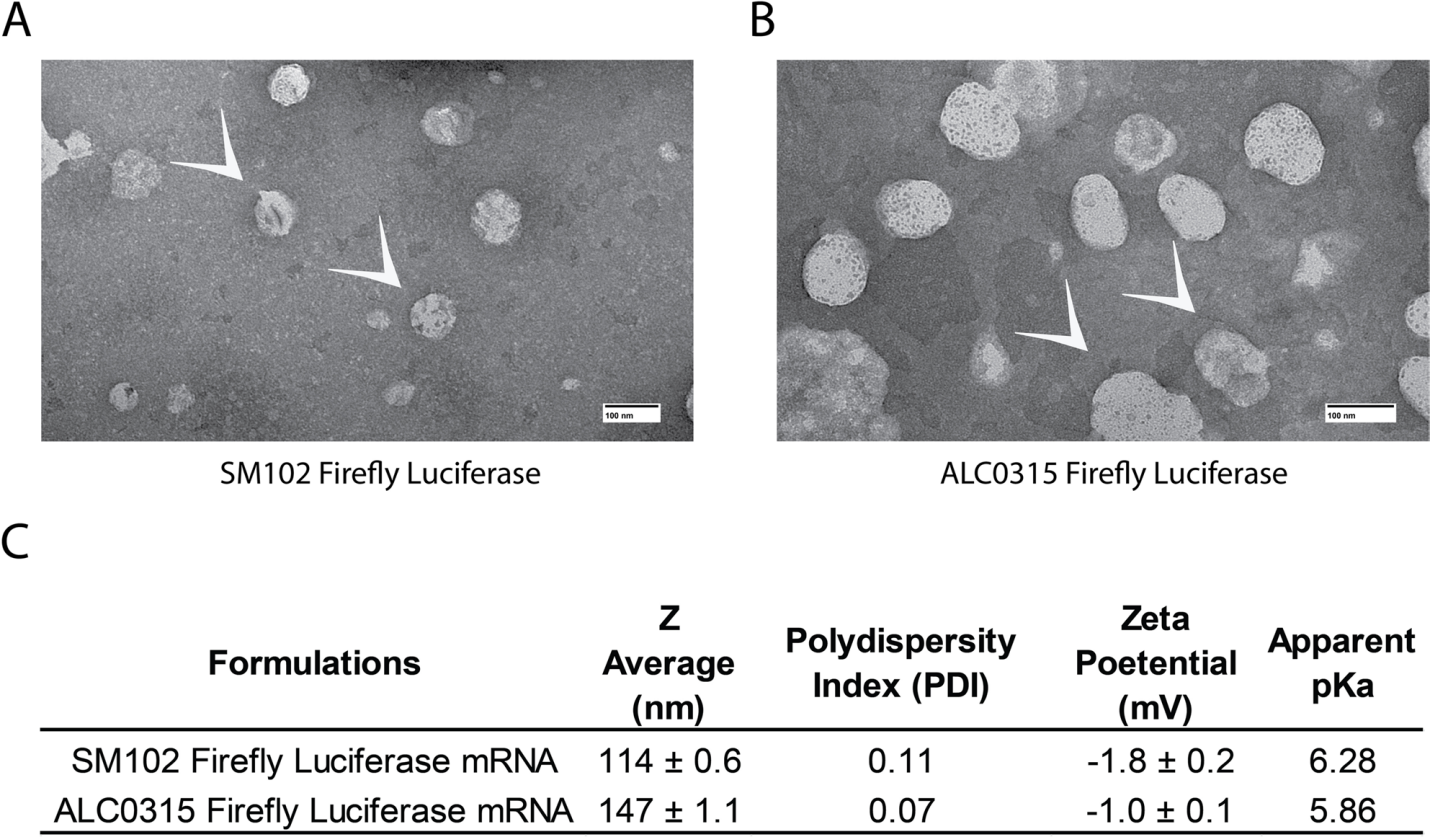
Fabrication and physicochemical characterization of firefly luciferase (FFLuc) mRNA-loaded SM102 and ALC0315 LNPs. **A** Transmission electron microscope (TEM) images of SM102 FFLuc mRNA LNPs and (**B**) ALC0315 FFLuc mRNA LNPs. The nanoparticles were indicated by white arrows. The Scale bar is 100 nm. **C** The table shows LNP’s physicochemical characteristics. Dynamic light scattering (DLS) was employed to measure particle size and polydispersity index (PDI) of the LNPs, with the hydrodynamic diameter reported as Z average in nanometers (nm). Electrophoretic light scattering (ELS) was used to determine the zeta potential of the LNPs, expressed in millivolts (mV). The apparent acid dissociation constant (pKa) of the LNPs was assessed using the TNS (6-p-Toluidino-2-naphthalenesulfonic acid) assay. All measurements are presented with standard deviation (SD) based on three replicates (*n* = 3)

We adapted our previously described abdominal window chamber (Liu et al. 2021a, 2021b), for imaging the inguinal lymph node. The procedure involves making a left paramedian incision approximately 1 cm long and attaching the abdominal window chamber to the skin with a purse-string suture. To position the lymph node against the imaging cover slip and secure it within the window chamber, we developed a backing (Fig. 1A). The implant was held in place by using single sutures between the backing and the eight holes of the window chamber. The lymph node was then observable within the specialized window chamber (Fig. 1B). Firefly luciferase mRNA (FFLuc) loaded ionizable LNPs were prepared using four different lipid components, including phospholipid, cholesterol, PEGylated-lipid, and ionizable lipid. Among ionizable lipids, we employed both SM102 and ALC0315, which are clinically approved for mRNA delivery (Han et al. 2021). LNPs were prepared through our previously developed flash nanoprecipitation technique using the confined impingement jet (CIJ) mixer (Misra et al. 2024). Transmission electron microscopy (TEM) confirms that both SM102 and ALC0315 LNPs encapsulating FFLuc mRNA exhibited spherical morphology (Fig. 2A and B). Dynamic light scattering (DLS) results showed LNPs had hydrodynamic diameters ranging from 114 to 147 nm (Fig. 2C) with polydispersity index (PDI) values below 0.3, suggesting monodispersity (Fig. 2C). Electrophoretic light scattering (ELS) measurements indicate LNPs exhibit neutral surface charge (Fig. 2C). Additionally, a 2-(p-toluidino)-6-naphthalene sulfonic acid (TNS) assay demonstrates apparent pKa values of 5.86 and 6.25 for ALC0315 and SM102 LNPs, respectively (Fig. 2C). The encapsulation efficiency of FFLuc mRNA was found to be greater than 95% in both SM102 and ALC0315 LNPs. All the physicochemical parameters of LNPs are in agreement with our earlier reported studies that showed precise mRNA delivery and generated desired antigen-specific immune responses in vivo (Misra et al. 2025).

**Fig. 3:**
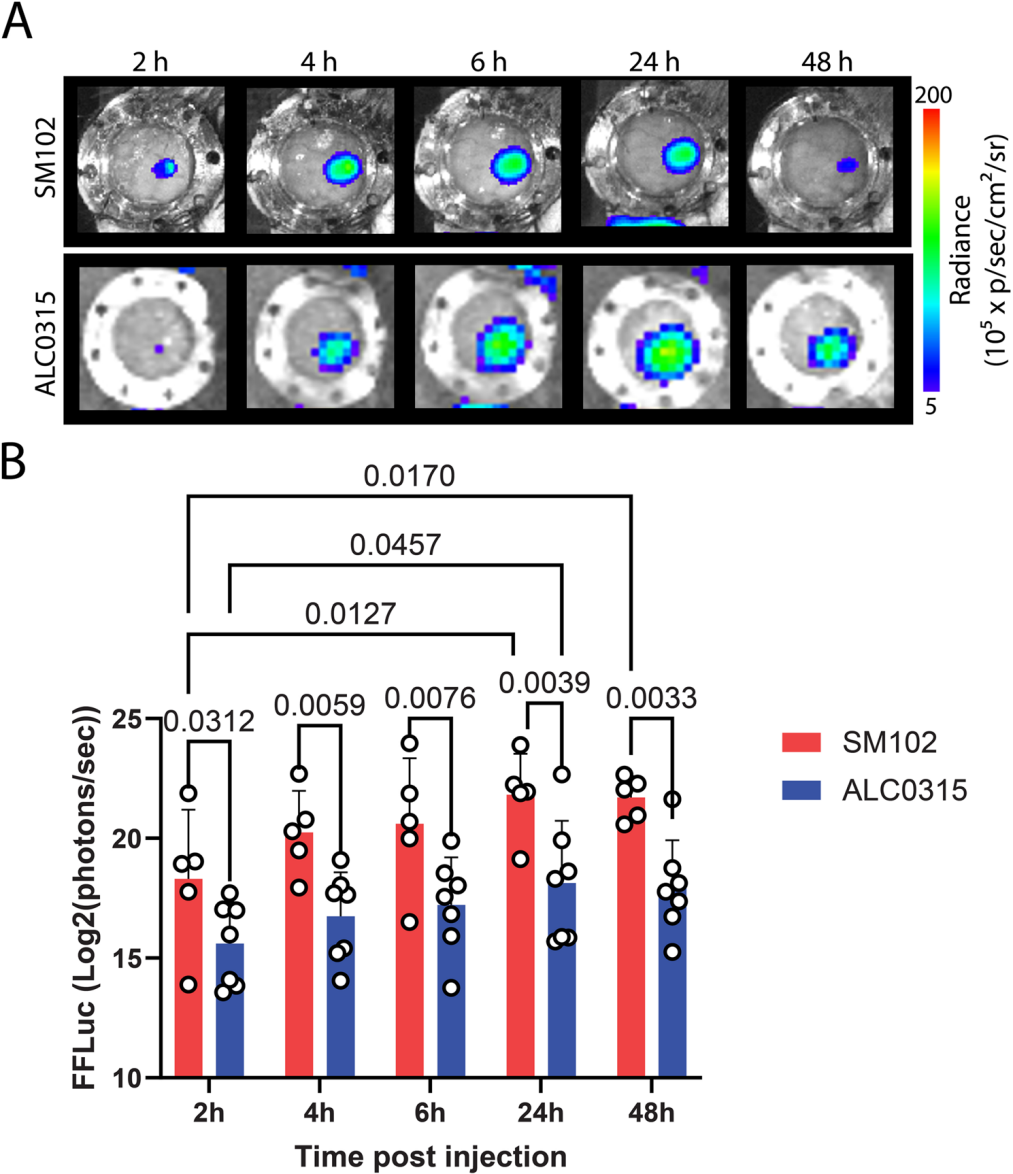
Real-time imaging of FFLuc bioluminescence in the lymph node following administration of FFLuc mRNA-loaded LNPs. **A** Representative FFLuc bioluminescence images over time and (**B**) corresponding quantification of FFLuc mRNA translation over time from SM102 and ALC0315 LNPs. Data shown as mean ± SD (*n* = 5–7 mice/group), pooled from two separate experiments. Significant differences between LNPs and each time point were determined by two-way ANOVA with Tukey’s multiple comparison test. *P* values are indicated in the graph

To track mRNA delivery of LNPs in the lymph node, we used FFLuc mRNA encoding firefly luciferase protein, which is measured by bioluminescence through IVIS imaging following the injection of D-Luciferin substrate. Following implantation of the lymph node window chamber, we intramuscularly injected (IM) FFLuc mRNA SM102 or ALC0315 LNPs and evaluated the real-time kinetics of mRNA delivery in the lymph nodes by bioluminescence imaging. Bioluminescence was observed in lymph nodes as soon as 2 h following administration of SM102 and ALC0315 LNPs, indicating the initiation of translation of mRNA (Fig. 3A). Interestingly, SM102 and ALC0315 LNPs showed a time-dependent increase in the signal until 24 h, which remained stable until the last time point tested, 48 h. FFLuc mRNA translation was significantly higher with SM102 LNPs than with ALC0315 LNPs at every time point tested (Fig. 3B). This is consistent with the literature and our previous report showing higher translational ability of SM102 LNPs as compared to ALC0315 LNPs (Zhang et al. 2023; Misra et al. 2024). This is mainly attributed to the higher pKa value of the SM102 ionizable lipid, which may allow optimal endosomal escape and greater cytoplasmic release of mRNA than the ALC0315 ionizable lipid. Whole-body imaging of inguinal lymph node window chamber-bearing mice revealed similar FFLuc bioluminescence in the inguinal lymph nodes and confirmed the expected hepatobiliary clearance of FFLuc mRNA for both SM-102 and ALC-0315 formulations (Fig. S1A). These results showcase the real-time translation ability of mRNA LNPs to protein in the lymph node and the retention kinetics of the translated protein at the site.

**Fig. 4:**
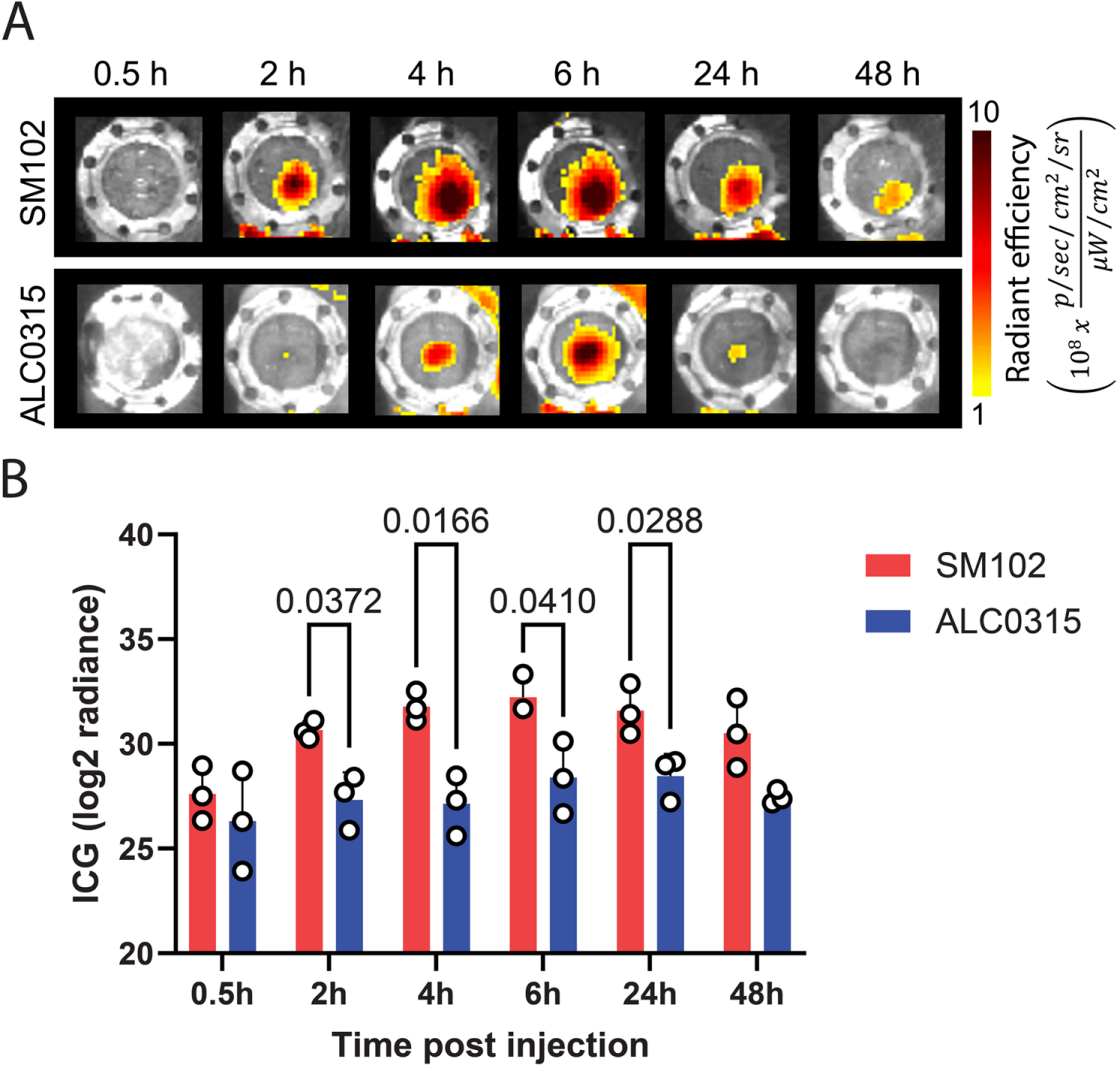
Real-time lymph node accumulation of ICG-loaded SM102 or ALC0315 LNPs. **A** Representative fluorescence images demonstrate ICG fluorescence over time in lymph nodes, represented as radiant efficiency, following IM administration of LNPs. **B** Corresponding quantification of radiance post-injection from the SM102 and ALC0315 LNPs. Data shown as mean ± SD (*n* = 3 mice/group). Significant differences between LNPs at each time point were determined by two-way ANOVA with Tukey’s multiple comparison test. P values are indicated in the graph

The bioluminescence signal observed in the earlier experiments allowed us to understand the mRNA delivery ability by LNPs. However, this may not be the right indicator of the rate of LNPs accumulation in the lymph node. Therefore, to further understand the inherent drainage kinetics of LNPs into the lymph nodes, we encapsulated LNPs with a near-infrared dye, indocyanine green (ICG), and measured the ICG fluorescence using IVIS imaging. ICG fluorescence measured as radiant efficiency showed minimal signal at 0.5 h following the IM dose in both SM102 and ALC0315 LNPs, with no significant differences (Fig. 4A&B). 2, 4, 6, and 24 h following the dose, LNPs demonstrated a drainage into lymph nodes from the injection site, with SM102 LNPs showing a significant accumulation as compared to ALC0315 LNPs (Fig. 4B). This data is in agreement with the luciferase signal observed in mRNA translation studies. SM102 and ALC0315 LNPs showed a peak ICG signal at 6 h with an apparent drop at the 48 h time point (Fig. 4). Whole body imaging of the lymph node window chamber-bearing mice showed similar ICG fluorescence in the inguinal lymph nodes. However, minimal hepatobiliary clearance of ICG was detected (Fig. S1B), which may be due to both the lower sensitivity of fluorescence imaging and the rapid clearance profile of ICG, together reducing the detectable hepatobiliary signal. We note that higher LNP accumulation in the lymph node may not always directly correlate with efficient mRNA translation and the induction of desired antigen-specific immune responses. It will be critical to corroborate mRNA translation with antigen presentation, immune cell activation, and induction of cytokines while optimizing the mRNA LNP vaccine formulation.

**Fig. 5:**
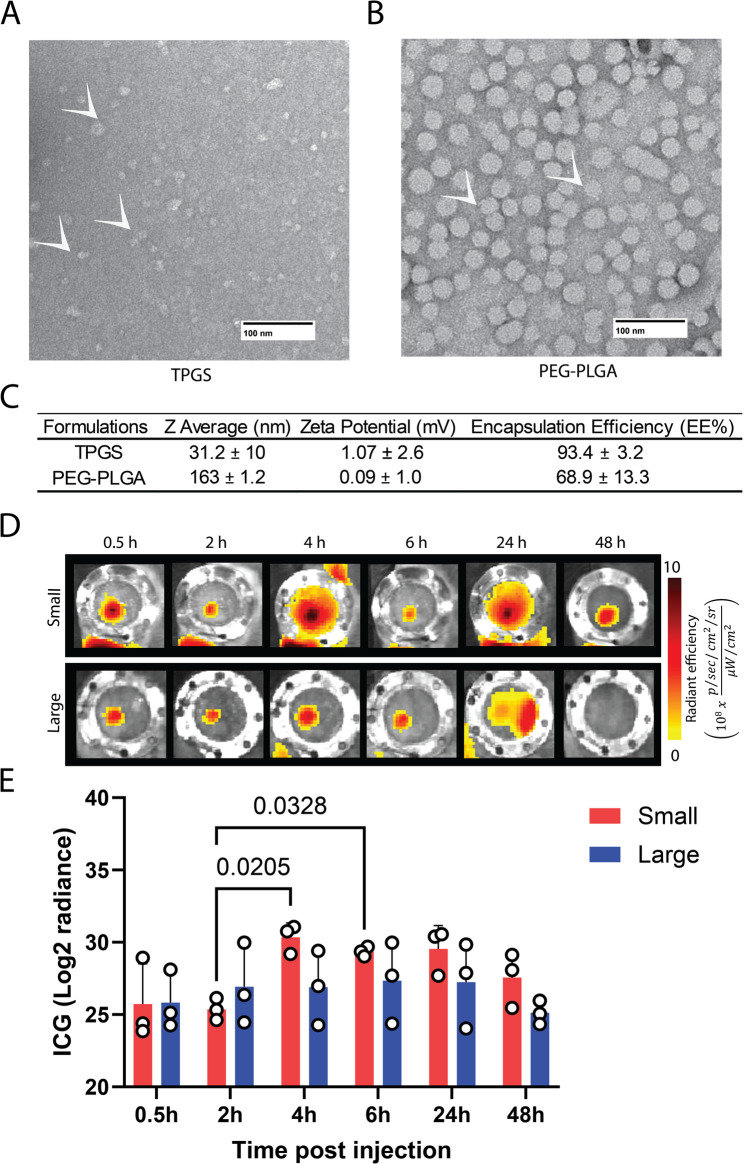
Fabrication and characterization of TPGS (small) and PEG-PLGA (Large) nanoparticles. **A** Transmission electron microscope (TEM) images of TPGS and (**B**) PEG-PLGA nanoparticles. The nanoparticles were indicated by white arrows. The Scale bar is 100 nm. **C** The table shows the nanoparticle’s physicochemical characteristics. Dynamic light scattering (DLS) was employed to measure the particle size of the LNPs, with the hydrodynamic diameter reported as Z average in nanometers (nm). Electrophoretic light scattering (ELS) was used to determine the zeta potential of the LNPs, expressed in millivolts (mV). Encapsulation efficiency of ICG was reported as percentage encapsulation. All measurements are presented as mean ± SD based on three replicates (*n* = 3). **D** Representative ICG fluorescence in lymph nodes over 48 h following intramuscular administration of small TPGS or large PEG-PLGA nanoparticles. **E** Corresponding quantification of radiance post-injection from the TPGS (small) and PEG-PLGA (Large) nanoparticles. All measurements are presented as mean ± SD based on three replicates (*n* = 3). Significant differences between LNP size and each time point were determined by two-way ANOVA with Tukey’s multiple comparison test. P values are indicated in the graph

Apart from LNPs, polymeric nanoparticles are widely studied vaccine carriers or adjuvants. Of note, it is well documented that polymeric nanoparticle drainage to lymph nodes is hugely dependent on their size. We next evaluated the drainage kinetics of polymeric nanoparticles with variable size to the LN through the chamber model following administration. To do this, we formulated polymeric nanoparticles with sizes below 50 nm and greater than 100 nm. TPGS, an esterified form of vitamin E succinate, with PEG 1000, or PEG-PLGA, were chosen to form smaller and larger nanoparticles, respectively. TPGS and PLGA are FDA-approved pharmaceutical excipients for various biomedical applications. Specifically, PLGA polymer, composed of lactic and glycolic acid esters, is a well-established platform for delivering vaccine components (Lü et al. 2009; Amjadi et al. 2013). We formulated TPGS and PEG-PLGA nanoparticles encapsulated with ICG-dye using the flash nanoprecipitation technique. TEM imaging confirms the spherical morphology of both the TPGS and PEG-PLGA nanoparticles (Fig. 5A and B). DLS analysis indicates the Z-average particle sizes of 31 and 163 nm for the TPGS and PEG-PLGA nanoparticles, respectively (Fig. 5C). ELS measurements indicate neutral surface charge for both TPGS and PEG-PLGA nanoparticles, indicating the presence of PEG on the surface of nanoparticles (Fig. 5C). ICG encapsulation efficiency in TPGS and PEG-PLGA nanoparticles was found to be 93.4 and 68.9%, respectively (Fig. 5C).

Following the morphological characterization, the in vivo accumulation within the lymph node of the TPGS nanoparticles compared to the PEG-PLGA nanoparticles loaded with ICG was evaluated using a lymph node window chamber (Fig. 5D). The smaller TPGS nanoparticles demonstrated a faster and enhanced accumulation into the lymph node as compared to the larger PEG-PLGA nanoparticles. Interestingly, TPGS nanoparticles signal peaked at 4 h where ICG radiance quantified at 4 and 6 h time points were significantly higher than the 2 h time point. This data may indicate that TPGS particles continually drain to lymph nodes due to their small size. Larger PEG-PLGA nanoparticles showed a variable accumulation rate in the lymph nodes with no significant difference between each time point tested, indicating a possibility of depot effect at the site of injection and accumulation relying on immune cell uptake (Fig. 5D&E). After 48 h, PEG-PLGA nanoparticles showed a lower signal of ICG, indicating a sign of clearance, while TPGS particles demonstrated a retention in the lymph node (Fig. 5D&E). The type of polymeric material and its interactions with immune cells may dictate the retention of particles at the lymph node site. It is important to note that our goal here is to validate the suitability of the lymph node window chamber model to study the lymphatic drainage of diverse nanoparticle types. Although the lymph node window chamber enables direct longitudinal imaging of nanoparticle accumulation into the lymph node, surgical implantation may alter the local microenvironment and potentially affect vascular permeability. While intramuscular injection favors lymphatic drainage, we cannot fully exclude minor contributions from altered vascular permeability introduced by the surgical preparation, which represents a limitation of the model. In addition, the ICG dye utilized in the study demonstrates rapid clearance in vivo, and any unprecedented release of the dye from nanoparticles may not accurately reflect the nanoparticle retention kinetics.

## Conclusion

We have successfully established the lymph node window chamber setup as a characterization tool for real-time evaluation of nanoparticle drainage and retention in the inguinal lymph node. Importantly, we demonstrated that lipid nanoparticles that are used in mRNA vaccine delivery can be evaluated for both the nanoparticle-driven drainage kinetics to lymph nodes and mRNA translation ability in lymphatic tissues. Furthermore, polymeric nanoparticles with variable size and material type can be optimized using this tool for vaccine applications. For our proof-of-concept, we have utilized fluorescence or bioluminescence markers to study nanoparticle distribution in the lymph node. It is important to note that here we only employed a model FFLuc mRNA, which may not be suitable to correlate translation of mRNA with the generation of antigen-specific cellular and humoral responses. Future studies warrant the co-encapsulation of disease-relevant vaccine components into nanoparticles to study the lymphatic drainage and immune responses generated. Overall, the characterization procedure showcased here may allow optimizing nanoparticle vaccines in the discovery phase with cost-efficiency, minimal variability, and reduced animal numbers.

## Supplementary Information

Supplementary Material 1.

## Data Availability

The datasets pertaining to all the experiments are obtainable upon request.
