# Supplementary material for: Real-time in vivo tracking of nanoparticulate vaccines in inguinal lymph nodes
Source: AAPS Open. 2026 Apr 27;12(1):23. doi: 10.1186/s41120-026-00163-5 (PMC13111494; doi:10.1186/s41120-026-00163-5)
Supplement: Supplementary file 1 — Supplementary Material 1. [file 41120_2026_163_MOESM1_ESM.docx]

Real-time in vivo tracking of nanoparticulate vaccines in inguinal lymph nodes

Bishal Misra^1^#, Kaitlyn M. Landreth^2,3^#, William H. Pentz^1,2^, Tracy W. Liu^2,3^, Sharan Bobbala^1^

^1^Department of Pharmaceutical Sciences, West Virginia University, Morgantown, WV 26506,

USA

^2^Department of Microbiology, Immunology and Cell Biology, West Virginia University, Morgantown, WV 26506, USA.

^3^West Virginia University Cancer Institute, Morgantown, WV 26506, USA

# Contributed equally

Corresponding authors:

Tracy W. Liu

Department of Microbiology, Immunology and Cell Biology, West Virginia University, Morgantown, WV 26506, USA.

Email: tracy.liu@hsc.wvu.edu

Phone: +1 304-293-3104

Sharan Bobbala

Department of Pharmaceutical Sciences, West Virginia University, Morgantown, WV 26506, USA

Email: sharan.bobbala@hsc.wvu.edu

Phone: +1 3042930279

Supplementary information


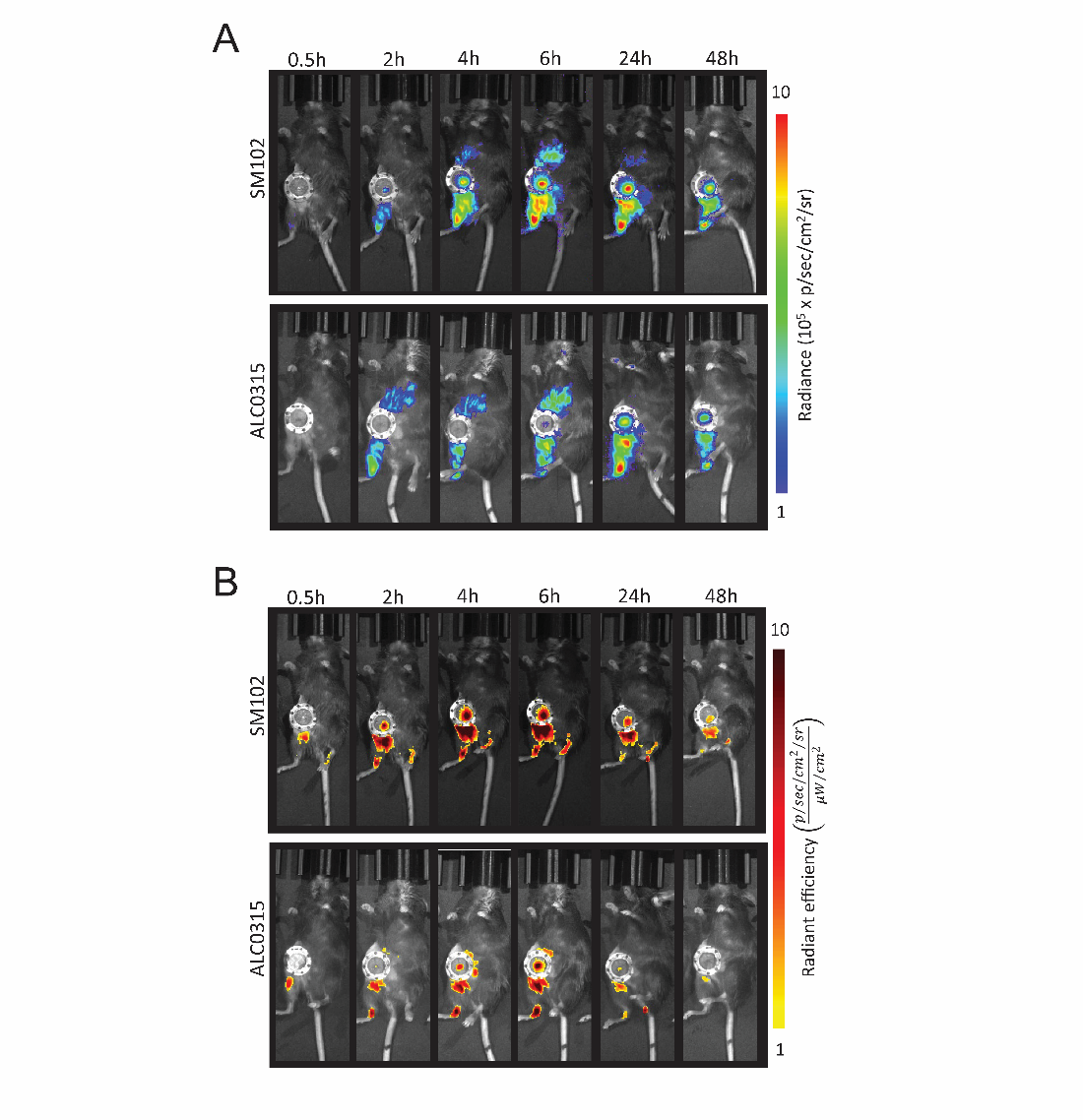


**Figure S1.** Real-time whole-body *in vivo* imaging of FFLuc mRNA or ICG LNPs. Representative (A) FFLuc bioluminescence images or (B) ICG fluorescence images over time for SM-102 compared to ALC-0315 LNPs.
